# Efficacy of a 2-Month Very Low-Calorie Ketogenic Diet (VLCKD) Compared to a Standard Low-Calorie Diet in Reducing Visceral and Liver Fat Accumulation in Patients With Obesity

**DOI:** 10.3389/fendo.2020.00607

**Published:** 2020-09-14

**Authors:** Guilherme Moura Cunha, German Guzman, Livia Lugarinho Correa De Mello, Barbara Trein, Luciana Spina, Isabela Bussade, Juliana Marques Prata, Ignacio Sajoux, Walmir Countinho

**Affiliations:** ^1^Department of Radiology, University of California, San Diego, La Jolla, CA, United States; ^2^Pronokal Group, Barcelona, Spain; ^3^Instituto Estadual de Diabetes e Endocrinologia Luiz Capriglione, Rio de Janeiro, Brazil; ^4^Rio de Janeiro, Rio de Janeiro, Brazil; ^5^Clínica Isabela Bussade, Rio de Janeiro, Brazil; ^6^Clinical Diagnostic Imaging (CDPI), Rio de Janeiro, Brazil

**Keywords:** very low-calorie ketogenic diet, visceral adipose tissue, NAFLD, liver PDFF, Pnk method, ketogenic diet

## Abstract

**Background:** Currently the treatment of non-alcoholic fatty liver disease (NAFLD) is based on weight loss through lifestyle changes, such as exercise combined with calorie-restricted dieting.

**Objectives:** To assess the effects of a commercially available weight loss program based on a very low-calorie ketogenic diet (VLCKD) on visceral adipose tissue (VAT) and liver fat content compared to a standard low-calorie (LC) diet. As a secondary aim, we evaluated the effect on liver stiffness measurements.

**Methods:** Open, randomized controlled, prospective pilot study. Patients were randomized and treated either with an LC or a VLCKD and received orientation and encouragement to physical activity equally for both groups. VAT, liver fat fraction, and liver stiffness were measured at baseline and after 2 months of treatment using magnetic resonance imaging. Paired *t*-tests were used for comparison of continuous variables between visits and unpaired test between groups. Categorical variables were compared using the χ^2^-test. Pearson correlation was used to assess the association between VAT, anthropometric measures, and hepatic fat fraction. A significance level of the results was established at *p* < 0.05.

**Results:** Thirty-nine patients (20 with VLCKD and 19 with LC) were evaluated at baseline and 2 months of intervention. Relative weight loss at 2 months was −9.59 ± 2.87% in the VLCKD group and −1.87 ± 2.4% in the LC group (*p* < 0.001). Mean reductions in VAT were −32.0 cm^2^ for VLCKD group and −12.58 cm^2^ for LC group (*p* < 0.05). Reductions in liver fat fraction were significantly more pronounced in the VLCKD group than in the LC group (4.77 vs. 0.79%; *p* < 0.005).

**Conclusion:** Patients undergoing a VLCKD achieved superior weight loss, with significant VAT and liver fat fraction reductions when compared to the standard LC diet. The weight loss and rapid mobilization of liver fat demonstrated with VLCKD could serve as an effective alternative for the treatment of NAFLD.

**Clinical Trial Registration:** ClinicalTrials.gov, identifier: NCT04322110.

## Introduction

More important than overall body weight, in overweight individuals and patients with obesity, the distribution of fat is strongly associated with the metabolic disturbances that lead to comorbidities (1, 2). Visceral adipose tissue (VAT) accumulation is associated with increased peripheral insulin resistance and often a systemic low-grade chronic inflammatory state, known as lipoinflammation (3–5). Similarly, liver fat accumulation, in this context called non-alcoholic fatty liver disease (NAFLD), is also associated with peripheral insulin resistance and a local inflammatory response, resulting in prolonged hepatocellular injury (6). Ultimately, the vicious cycle installed between these fat depots and secondary metabolic derangements may lead to organ damage and high cardiovascular risk in individuals with obesity.

NAFLD is one of the most common causes of chronic liver disease worldwide, with a prevalence still in a rise proportionally with the rise in obesity, sedentary lifestyle, unhealthy dietary pattern, and metabolic syndrome (7). In a proportion of these patients, the presence of chronic hepatic inflammation leads to more advanced forms of disease, i.e., non-alcoholic steatohepatitis and fibrosis, with the potential of liver failure and increased risk of hepatocellular carcinoma (8). Currently, the first-line treatment of NAFLD consists of weight loss and lifestyle modifications such as physical exercise and dietary regimens based on calorie restriction (9, 10). However, lifestyle interventions including standard low-calorie (LC) diets and exercise often do not achieve significant enough weight loss to reverse the fat accumulation in the liver (6, 11). To date, there is still a paucity of effective targeted or specific therapies for NAFLD (6, 12). Alternatively, bariatric surgery promotes effective weight loss capable of improving fat deposition in the liver and in the visceral compartment, although with some risk of surgical complications, particularly in patients with comorbidities (13–15). Very LC ketogenic diet (VLCKD) has been proposed as an effective weight loss intervention potentially suitable for the treatment of NAFLD and for the reduction of VAT, which may be beneficial to reduce the state of insulin resistance and end-organ damage (16–18).

Magnetic resonance imaging (MRI) has been shown as an accurate method for measuring VAT and liver fat in clinical practice and in clinical trials (19–21). It also allows the estimation of liver stiffness, a non-invasive biomarker that correlates to liver inflammation and fibrosis (22, 23). The aim of this study was to assess and compare the effects of a commercially available program for weight loss that includes VLCKD and a standard LC diet on VAT and liver fat content using MRI. As a secondary aim, we evaluated the effect of both interventions on liver stiffness measurements.

## Materials and Methods

This was an open-label, randomized, two-arm, parallel-group controlled study approved by and in accordance to the recommendations of the local ethics committee; all patients provided a written consent confirming the willingness to participate in the study.

### Subjects

We prospectively recruited consecutive patients clinically referred for weight loss treatment at the department of obesity, eating disorders and metabology of the Instituto Estadual de Diabetes e Endocrinologia (IEDE, Rio de Janeiro, Brazil). Subjects' demographics and anthropometric laboratory measurements were collected. Inclusion criteria were (i) age 18 years or older, (ii) body mass index (BMI) higher than 30 kg/m^2^, and (iii) willingness and ability to complete all research procedures. The exclusion criteria were (i) any formal contraindication to one of the weight loss interventions, including diabetes diagnosed at any phase of the study; (ii) inability to complete the dietetic and/or behavior modification programs; (iii) history or suspicion of alcohol abuse based on laboratory findings and clinical history; and (iv) contraindications to MRI. While not in the scope of the study, liver function tests [aspartate aminotransferase (AST), alanine aminotransferase (ALT), alkaline phosphatase, γ-glutamyl transpeptidase (GGT), bilirubin, albumin, platelets], as well as kidney function tests, were collected at baseline and follow-up to monitor for indirect signs of hepatocellular dysfunction.

### Interventions

Patients were randomized 1:1 in two groups to receive either a commercially available VLCKD or a standard-of-care LC diet for weight loss purposes. Random allocation was done according to a computer-generated randomization list. All study participants underwent periodic clinical assessment by a specialized endocrinologist through the course of the study. Aiming for congruency between both groups, patients were maintained under the correspondent weight-loss regimens for the same period of time (2 months) when the follow-up MRI was performed. Orientations on lifestyle changes, adequate dietary habits, and physical activity were provided by expert support staff comprising dietitians and physical trainers to both groups at the initial visit.

### Low-Calorie Diet

The standard LC diet was an equilibrated diet that had a caloric value 15% below the total metabolic expenditure of each individual. The total metabolic expenditure was calculated from the basal metabolic expenditure (based on the formula Food and Agriculture Organization/World Health Organization/United Nations)^1^ multiplied by the coefficient of activity of each participant. The calories provided to this group ranged between 1,400 and 1,800 kcal/day. The ratio of macronutrients provided was 45–55% carbohydrates, 15–25% proteins, and 25–35% fat in addition to a recommended intake of 20–40 g/day of fiber in the form of vegetables and fruits.

### Very Low-Calorie Ketogenic Diet

**Figure 1:**
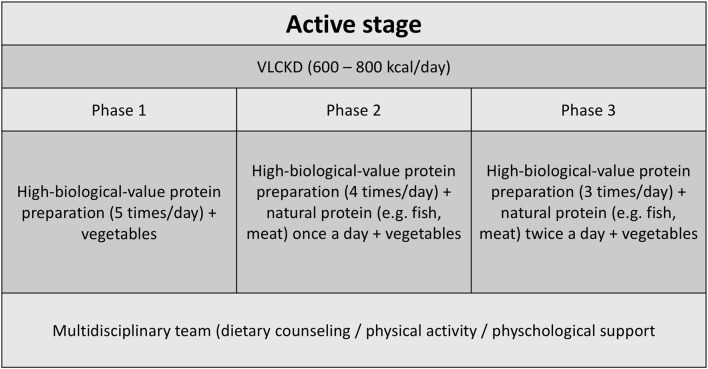
Active phase dietary counseling for patients undergoing the VLCKD method.

The VLCKD group followed a diet according to the first stage of a commercial weight loss program (Pronokal® method) (24) based on a high biological value protein preparation. During the 2 months of the study, patients were kept in a ketogenic stage (“active stage”) of the method, which consists of a very LC diet (600–800 kcal/day) and low in carbohydrates (<50 g daily from vegetables) and lipids (only 10 g of olive oil per day) (Figure 1). The amount of high-biological-value proteins ranged between 0.8 and 1.2 g per each kilogram of ideal body weight, to ensure meeting the minimal body requirements and to prevent the loss of lean mass (16).^2^ Throughout the ketogenic phase, supplements of vitamins and minerals were provided in accordance to international recommendations (Supplementary Materials).

### Outcome Measurements

Anthropometric measurements were recorded at baseline and follow-up. Abdominal MRI was performed at baseline and at 2 months' follow-up treatment using an abbreviated protocol (25) for the specific purpose of this study. Patients were imaged in a 1.5-T MR scanner (GE 450 W; GE Medical Systems, Milwaukee, WI, USA) in the supine position without the use of intravenous contrast administration. All image analysis was performed by a fellowship-trained abdominal radiologist with 10 years of experience who was blinded to patients' clinical information and randomization.

### Visceral Adipose Tissue Area

Manual VAT segmentation was performed on water images obtained with an MRI chemical-shift sequence to ensure maximal contrast between fat and non-fatty tissues. The VAT area in the axial slice at the level of the third/fourth lumbar intervertebral disk (L3–L4) was measured. This level was chosen in accordance to the previous descriptions in the literature which report a higher correlation with total visceral volume and its variations (21, 26). VAT measurements were provided in square centimeters (cm^2^).

### Liver Proton Density Fat Fraction Measurements

To estimate liver fat content, measurements of liver proton density fat fraction (PDFF) were performed using a multigradient-echo confounder-corrected chemical shift–encoded sequence (IDEAL-IQ®). Five-square-centimeter electronic regions of interest (ROIs) were drawn over the right liver lobe on the MRI-PDFF parametric maps, avoiding major vessels, the gallbladder fossa, or focal lesions. PDFF is expressed in percentile as a non-invasive biomarker of liver steatosis (19). Liver steatosis was defined as PDFF >5.4% in accordance to reports investigating patients with obesity (20).

### Liver Stiffness

A commercially available standard liver two-dimensional gradient-echo MR elastography (MRE) sequence (MR-Touch® General Electric, Milwaukee, WI, USA) was used to acquire stiffness maps at the midportion of the liver, in correspondence to the PDFF sections. MRE imaging parameters are described elsewhere (25). ROIs were used to estimate mean liver stiffness values over the right liver lobe in correspondence to the PDFF measurements. Stiffness values are expressed in mean kilopascals (kPa). Liver stiffness values below 2.8 kPa were considered as absence of fibrosis, values between 2.8 and 4.1 kPa indicative of mild fibrosis, and higher than 4.15 kPa considered a sign of advanced fibrosis (22, 27, 28).

### Statistical Analysis

To summarize the demographic and clinical characteristics of the cohort, a descriptive analysis was performed using measurements of central tendency and dispersion (mean, median; min–max). For this exploratory pilot study to prove a concept, we did not perform a power analysis to estimate sample sizes. Study data distribution was assessed for normality using the Shapiro–Wilk test, and study results reported as means and standard deviations (SDs). All comparisons from baseline to 2 months of follow-up have been made on “completers.” For comparison of the variables [overall weight, BMI, waist circumference (WC), VAT area, hepatic fat fraction, and liver stiffness], a Student *t*-test was used for the continuous variables using an unpaired test for the comparison between groups and a paired test for the comparison between visits. The comparisons between groups on the categorical variables were performed using the χ^2^-test. To evaluate the possible correlation between VAT, anthropometric measures, and hepatic fat fraction, the Pearson correlation coefficient was estimated. All analysis was carried out on the data set using the available information with criteria of intention to treat. A significance level of the results was established at *p* < 0.05.

## Results

**Figure 2:**
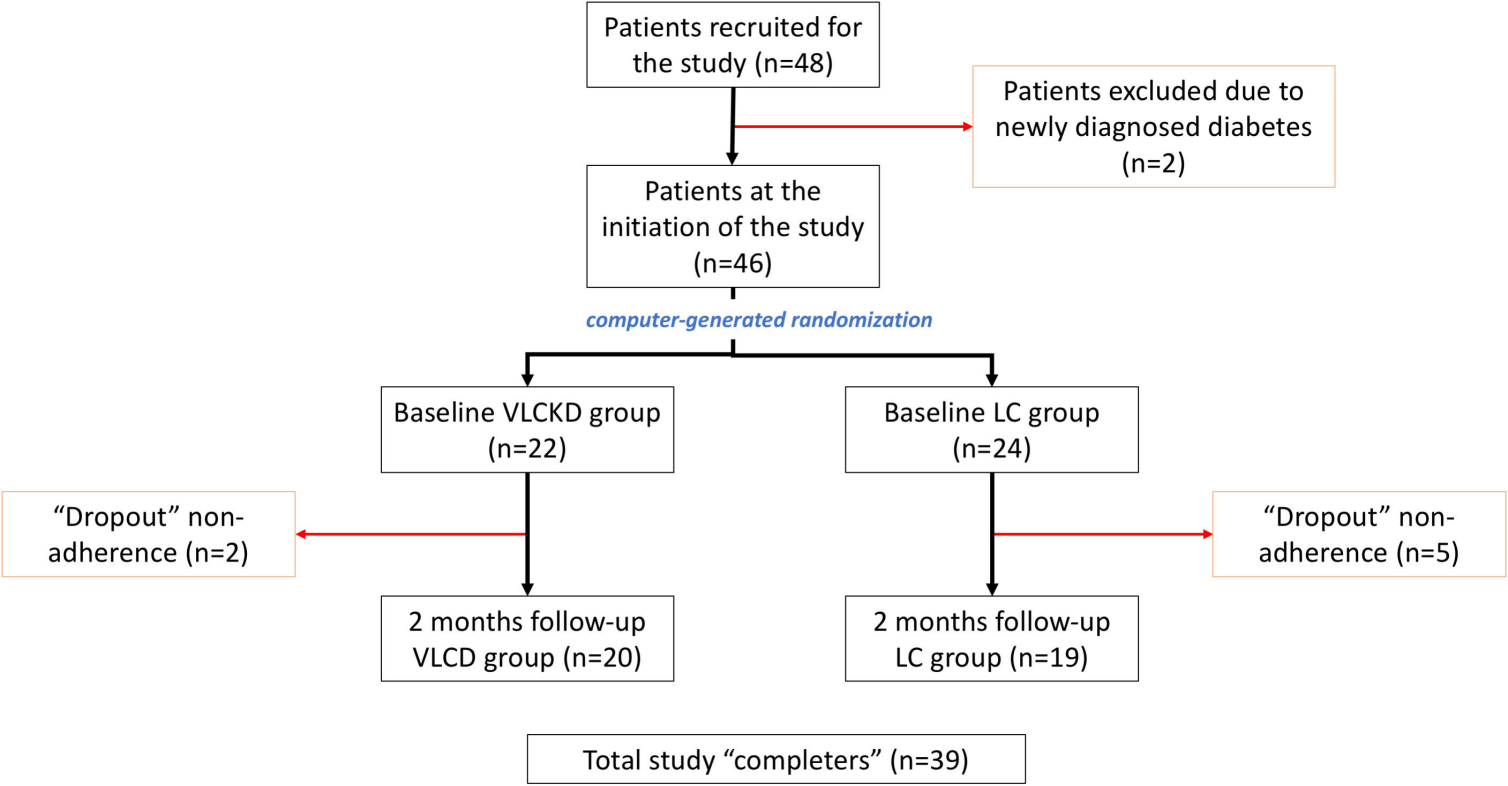
Study diagram describing the included population and groups.

From December 2016 to May 2018, a total of 48 patients were prospectively recruited for the study. After applying the exclusion criteria, two patients were excluded because of recent-onset diabetes diagnosed after recruitment. Hence, the initial study cohort consisted of 46 patients, 22 in the VLCKD group and 24 in the LC group (Figure 2). At baseline, the cohort characteristics were as follows: 8 (17.4%) men and 38 (82.6%) women; age: mean 40.3 ± SD 11.3 (range = 18–59) years old; body weight: mean 95.6 ± SD 14.3 (median = 91.5; range = 70.2–133.9) kg; and BMI: mean 35.7 ± SD 4.3 (median = 35.3; range = 30.0–47.3) kg/m^2^, without significant differences between both groups. During the course of the study, seven patients dropped out because of non-adherence to treatment (five from the LC group and two from the VLCKD group). Ultimately, at 2 months follow-up, 39 patients (20 in the VLCKD group and 19 in the LC group) were reassessed (“completers”) (Table 1).

**Table 1:** Study population characteristics (“completers” only).

	**VLCK diet** **(*n* = 20)**	**LC diet** **(*n* = 19)**	***p***
	**Mean ± SD**	**Mean ± SD**	
Weight (kg)	99.55 ± 13.24	93.01 ± 13.28	0.1323^a^
BMI (kg/m^2^)	37.1 ± 4.28	34.84 ± 4.33	0.1092^a^
Waist circumference (cm)	111 ± 9.77	108.53 ± 10.35	0.4474^a^
% Fat fraction (PDFF)^*^	10.01 ± 6.74	9.03 ± 6.8	0.6554^a^
Presence of steatosis^**^	14	12	0.4547^b^
Liver stiffness	2.0 ± 0.32	1.85 ± 0.32	0.2912^a^

* *PDFF: liver proton density fat fraction*.

** *Liver PDFF >5.4%*.

*p-values: ^a^Comparison between groups, Student unpaired-sample t-test*.

b *Comparison between groups, χ^2^-test*.

### Anthropometric Measures

At 2 months, absolute weight loss was significantly more pronounced in the VLCKD group than in the LC group (−9.7 ± 3.9 kg vs. −1.67 ± 2.2 kg; *p* < 0.0001). Relative weight loss was also significantly more pronounced in the VLCKD group (−9.59% ± 2.87% vs. −1.87% ± 2.4%; *p* < 0.0001). To the same extent, the reduction in BMI and WC in the VLCKD group was significantly greater than in the LC group (BMI: −3.56 ± 1.3 kg/m^2^ vs. −0.71 ± 0.9 kg/m^2^; *p* < 0.0001; WC: −8.0 ± 3.9 cm vs. −3.0 ± 2.9 cm; *p* < 0.0001) (Table 2).

**Table 2:** Anthropometric measurements during the follow-up period.

	**Total (completers)**	**VLCK diet**	**LC diet**	***p*^a^**
	**(*n* = 39)**	**(*n* = 20)**	**(*n* = 19)**	
	**Mean ± SD (95% CI)**	**Mean ± SD (95% CI)**	**Mean ± SD (95% CI)**	
**Weight (kg)**				
Baseline	96.36 ± 13.5	99.55 ± 13.24	93.01 ± 13.28	0.1323
2 months	90.57 ± 12.17	89.85 ± 10.94	91.34 ± 13.61	0.7084
Difference	−5.79 ± 5.14	−9.70 ± 3.88	−1.67 ± 2.22	<0.0001
*p*^b^	—	<0.0001	0.0041	
**BMI (kg/m**^**2**^**)**				
Baseline	36 ± 4.4	37.1 ± 4.28	34.84 ± 4.33	0.1092
2 months	33.83 ± 4.07	33.55 ± 3.73	34.13 ± 4.49	0.659
Difference	−2.17 ± 1.85	−3.56 ± 1.34	−0.71 ± 0.96	<0.0001
*p*^b^	—	<0.0001	0.005	
**Waist circumference (cm)**				
Baseline	109.79 ± 10.00	111 ± 9.77	108.53 ± 10.35	0.4474
2 months	104.21 ± 9.94	103.00 ± 9.68	105.47 ± 10.31	0.4446
Difference	−5.59 ± 4.26	−8.00 ± 3.96	−3.05 ± 2.91	<0.0001
*p*^b^	—	<0.0001	0.0002	

a *p for comparisons between groups among completers. Student unpaired-sample t-test*.

b *p for comparisons between baseline and 2 months' follow-up for each group. Student paired-sample t-test*.

### Biochemical Parameters

The biochemical parameters showed no significant changes in any group except for a decrease in AST (from 22.88 to 18.93; *p* < 0.05), HbA_1c_ (from 5.52 to 5.41; *p* < 0.05), and uric acid (from 5.38 to 5.10; *p* < 0.05) in the VLCKD group and a decrease in total cholesterol in both groups (Table 3).

**Table 3:** Biochemical parameters during the follow-up period.

	**Total**	**VLCK diet**	**LC diet**	
	**(*n* = 39)**	**(*n* = 20)**	**(*n* = 19)**	
	**Mean ± SD (95% CI)**	**Mean ± SD (95% CI)**	**Mean ± SD (95% CI)**	***p*^a^**
**Glucose**				
Baseline	86.90 (7.87)	84.82 (7.15)	88.38 (8.16)	0.1569
2 months	88.91 (8.91)	85.67 (7.59)	91.61 (9.22)	0.055
*p*^b^	—	0.6843	0.0922	
**HbA**_**1c**_				
Baseline	5.53 (0.37)	5.52 (0.42)	5.53 (0.34)	0.9624
2 months	5.38 (0.33)	5.41 (0.36)	5.37 (0.31)	0.7361
*p*^b^	—	0.0412	0.3051	
**Insulin**				
Baseline	14.50 (6.22)	12.85 (6.20)	15.67 (6.09)	0.155
2 months	13.92 (7.37)	12.70 (7.45)	14.99 (7.35)	0.3883
*p*^b^	—	0.4881	0.4811	
**HOMA-IR**				
Baseline	3.14 (1.45)	2.72 (1.40)	3.44 (1.44)	0.1148
2 months	3.09 (1.77)	2.70 (1.58)	3.44 (1.90)	0.2441
*p*^b^	—	0.5518	0.7466	
**Urea**				
Baseline	26.07 (7.28)	24.47 (6.32)	27.21 (7.82)	0.2403
2 months	26.76 (7.85)	28.27 (8.99)	25.50 (6.77)	0.3211
*p*^b^	—	0.079	0.6731	
**Creatinine**				
Baseline	0.80 (0.16)	0.77 (0.08)	0.82 (0.20)	0.3461
2 months	0.76 (0.14)	0.75 (0.09)	0.76 (0.18)	0.8197
*p*^b^	—	0.5306	0.1013	
**Glutaminic Oxaloacetic Transaminase (AST)**				
Baseline	21.12 (7.28)	22.88 (8.54)	19.88 (6.12)	0.1963
2 months	17.90 (3.86)	18.93 (3.92)	16.94 (3.66)	0.1533
*p*^b^	—	0.0382	0.1952	
**Glutaminic Pyruvic Transaminanse (ALT)**				
Baseline	24.46 (14.11)	26.12 (15.43)	23.29 (13.30)	0.5342
2 months	21.39 (10.48)	22.73 (11.26)	20.13 (9.90)	0.4981
*p*^b^	—	0.2246	0.3808	
**Alkaline Phosphatase**				
Baseline	77.34 (21.12)	79.88 (19.03)	75.54 (22.72)	0.5237
2 months	78.70 (20.68)	80.67 (22.02)	76.73 (19.82)	0.6111
*p*^b^	—	0.5537	0.5195	
**GGT**				
Baseline	60.63 (90.50)	85.06 (135.48)	43.33 (26.87)	0.1481
2 months	45.00 (71.61)	54.73 (100.75)	35.27 (16.35)	0.4663
*p*^b^	—	0.1637	0.0797	
**Triglycerides**				
Baseline	128.00 (62.98)	133.76 (62.36)	123.92 (64.42)	0.6279
2 months	112.06 (50.69)	114.67 (55.03)	109.89 (48.29)	0.7923
*p*^b^	—	0.0998	0.2882	
**Total cholesterol**				
Baseline	185.83 (34.73)	187.18 (43.97)	184.88 (27.39)	0.8374
2 months	172.18 (39.01)	173.60 (52.14)	171.00 (24.94)	0.8523
*p*^b^	—	0.0477	0.0036	
**High-density Lipoprotein**				
Baseline	51.90 (12.62)	50.41 (9.41)	52.96 (14.58)	0.5313
2 months	49.33 (11.76)	47.87 (7.17)	50.56 (14.65)	0.5218
*p*^b^	—	0.4027	0.1036	
**Low-density Lipoprotein**				
Baseline	108.34 (25.72)	110.12 (33.96)	107.08 (18.55)	0.7148
2 months	101.03 (31.38)	103.27 (41.16)	99.17 (21.22)	0.7149
*p*^b^	—	0.2159	0.0651	
**Uric acid**				
Baseline	5.31 (1.30)	5.38 (1.19)	5.27 (1.39)	0.7825
2 months	5.03 (0.96)	5.10 (1.04)	4.98 (0.91)	0.7218
*p*^b^	—	0.0229	0.8341	

a *p for comparisons between groups among completers. Student unpaired-sample t-test*.

b *p for comparisons between baseline and 2 months' follow-up for each group. Student paired-sample t-test*.

### Visceral Adipose Tissue

**Figure 3:**
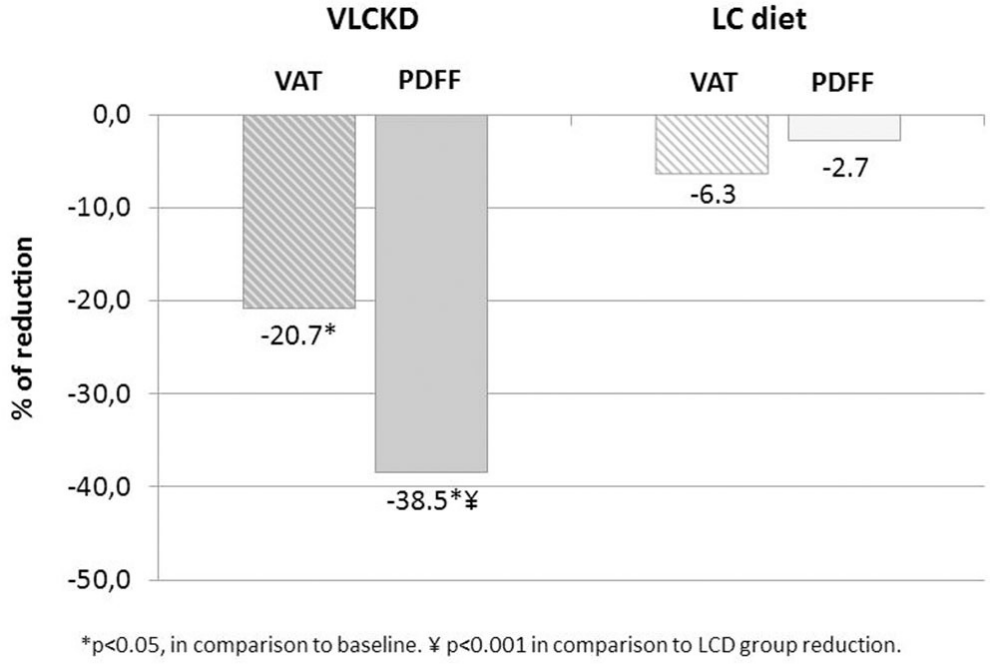
Reduction in VAT and liver fat content assessed by PDFF between the two study groups.

From baseline to 2 months' follow-up, a significant reduction in VAT was seen in the VLCKD group (170.8 ± 58.0 vs. 131.5 ± 47.7 cm^2^; *p* < 0.05) but not in the LC group (134.6 ± 76.3 vs. 122.05 ± 77.5 cm^2^; *p* > 0.1). The relative reduction of VAT was higher in the VLCKD group compared to the LC group, although there was only a trend toward statistical significance (−21.47% ± 19.1% vs. −6.33% ± 28.9%; *p* = 0.06) (Figure 3).

### Liver Proton Density Fat Fraction

**Figure 4:**
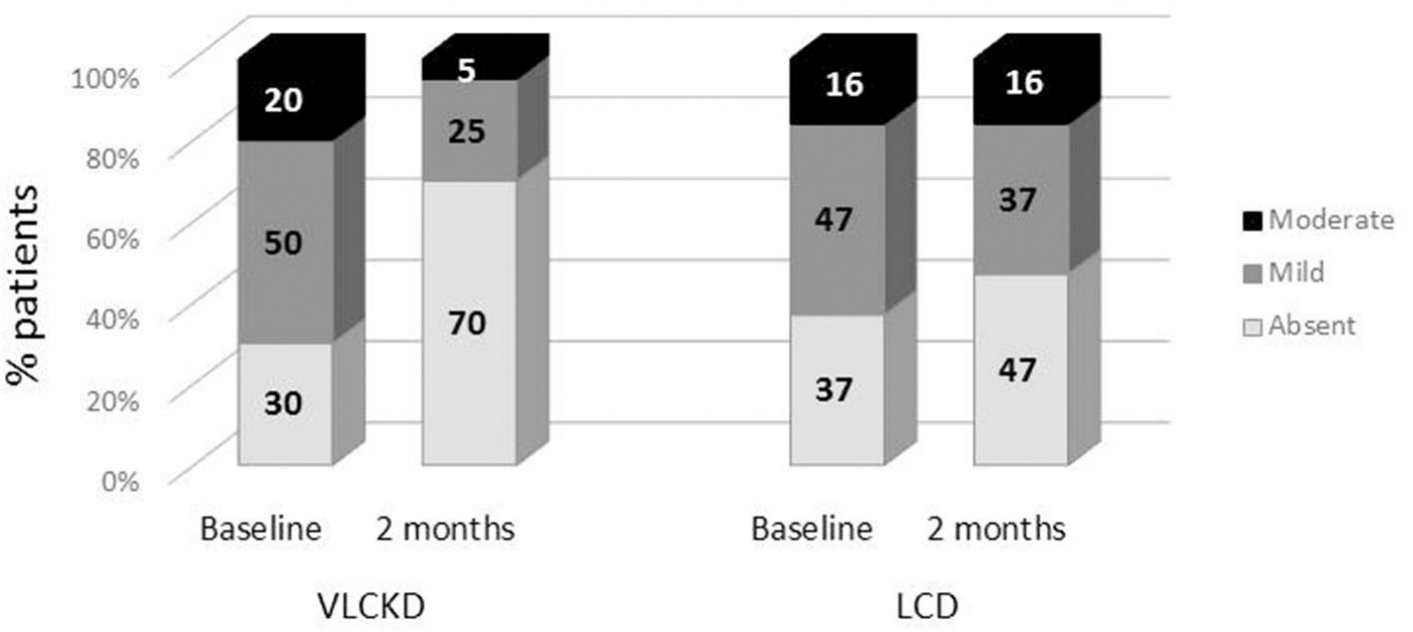
Comparison of liver steatosis between VLCKD and LCD groups at baseline and follow-up.

The VLCKD resulted in a significant decrease in PDFF values compared to the LC diet (mean ± SD = −4.77 ± 4.26 vs. −0.79 ± 1.76 *p* = 0.0006; mean relative change = −38.5 vs. −2.7%; *p* < 0.0001) (Figure 3). The prevalence of liver steatosis (PDFF >5.4%) in the intervention group decreased from 70.0% (14/20) to 30.0% (6/20) and in the LC group from 63.2% (12/19) to 52.6% (10/19) (Figure 4). A significant decrease in steatosis grades was also seen in VLCKD compared to the LC group (*p* = 0.0351) (Table 4).

**Table 4:** Assessment of liver fat fraction during the follow-up period.

	**Total ± completers)**	**VLCK diet**	**LC diet**	
	**(*n* = 39)**	**(*n* = 20)**	**(*n* = 19)**	
% Fat fraction (PDFF)^**^	**Mean ± SD (95% CI)**	**Mean ± SD (95% CI)**	**Mean ± SD (95% CI)**	***p*^a^**
				
Baseline	9.53 ± 6.7 (7.5)	10.01 ± 6.74 (7.9)	9.03 ± 6.8 (6.2)	0.6554
2 months	6.70 ± 5.06 (4.3)	5.24 ± 3.81 (3.8)	8.24 ± 5.82 (6.2)	0.0634
Difference	−2.83 ± 3.82 (−1.8)	−4.77 ± 4.26 (−3.4)	−0.79 ± 1.76 (−0.5)	0.0006
*p*^b^	—	<0.0001	0.0651	
Liver steatosis^*^	***n*** **(%)**	***n*** **(%)**	***n*** **(%)**	***p*^c^**
Baseline	26(66.7%)	14(70%)	12(63.2%)	0.4547
2 months	16 (41.0%)	6 (30.0%)	10 (52.6%)	0.1509
Improvement	10 (25.6%)	8 (40.0%)	2 (10.5%)	0.0351
No improvement	29 (74.4%)	12 (60.0%)	17 (89.5%)	

* *Liver steatosis = PDFF >5.4%*.

** *PDFF = liver proton density fat fraction*.

a *p-value for comparisons between groups among completers. Student unpaired-sample t-test*.

b *p-value for comparisons between baseline and 2 months' follow-up for each group. Student paired-sample t-test*.

c *p-value for comparisons between groups among completers χ^2^-test*.

### Liver Stiffness

At baseline, none of the patients in our study showed signs of liver fibrosis, and no differences in mean liver stiffness values were seen between groups (2.0 ± 0.32 kPa in the VLCKD group and 1.85 ± 0.32 kPa in the LC group; *p* = 0.2912). Of note, at baseline, two patients in the LC group and one in the VLCKD group had borderline high liver stiffness values (2.5, 2.6, and 2.6 kPa). In both groups, no significant changes in liver stiffness occurred from baseline to follow-up.

### Relationships Between Weight, VAT, and PDFF

Overall, a positive relationship between weight and VAT was confirmed, at baseline (*r* = 0.474; *p* < 0.05) and at 2 months (*r* = 0.609; *p* < 0.05), although only the VLCKD group showed a positive correlation between VAT loss and weight at 2 months. Regarding the relationship between VAT and liver PDFF changes, the Pearson correlation showed significant correlation between VAT and relative reduction in PDFF at 2 months only in the LC group (*r* = 0.4622; *p* < 0.05), but not in the VLCKD group (*r* = 0.2996; *p* > 0.05). The percentage reduction was greater for VAT than for PDFF in the LC group and conversely was greater for PDFF than for VAT in the VLCKD group (Figure 3).

## Discussion

This study aimed to prospectively evaluate the effects of a VLCKD on VAT and liver fat fraction measured by MRI compared to a standard LC diet. Secondarily, we performed a longitudinal assessment of liver stiffness, a surrogate of inflammation and fibrosis in both groups. Not only did we find expected higher weight loss and VAT reductions in patients undergoing the program with VLCKD than in patients undergoing an LC diet, but we also found a significantly higher decrease in liver fat content in the former population. No patient showed changes in liver stiffness during the interventions. Our results corroborate that the therapeutic implementation of VLCKDs can result in a faster mobilization of liver fat and VAT than current standard weight-loss regimens.

Our cohort comprised adult individuals from the general population undergoing weight loss treatment for clinical care. Not all patients had liver steatosis, with only 67.4% showing a PDFF >5.4% at baseline, mirroring the prevalence of liver steatosis in populations with obesity (29). In our study, greater VAT and PDFF reductions were seen in patients treated with a VLCKD in comparison to patients undergoing an LC diet. At 2 months, only in the LC group a high correlation between VAT and PDFF persisted, whereas this correlation was lost in the VLCKD group because of the higher reduction in PDFF relative to VAT. This particular finding contributes to confirm the hypothesis that the mobilization of liver fat occurs faster than in other compartments in patients undergoing VLCKD interventions (13, 17). Although one could argue that these results relate to the inequality in calorie intake between VLCKD and conventional LC diets, the differences regarding the correlation between VAT and PDFF at baseline and at 2 months between groups may indicate differently. Studies have shown that the fast reduction in liver fat is probably more related to the ketogenic state than to overall calorie restriction (17), and insulin resistance has been suggested as a key mechanism in this process (30). In a prior study, Luukkonen et al. (17) have described a high rate of liver triglycerides hydrolysis during the increased hepatic production of ketones, as serum insulin concentrations, endogenous glucose production, and hepatic insulin resistance decrease in patients undergoing VLCKD. In our study, however, this relationship was not confirmed as differences in reduction of Homeostatic Model Assessment of Insulin Resistance (HOMA-IR) between both groups failed to reach statistical differences. It is likely that the lack of statistical significance in our study is related to the small sample size, however conceivable that the reduction in VAT and liver fat fraction in such a short term in patients undergoing VLCKD is also related to additional metabolic changes. Most studies to date examining the relationship between NAFLD and insulin resistance are cross-sectional in design, have small sample sizes, or use rat models (17, 31–38), and hence, future longitudinal clinical studies with larger cohorts should be performed to validate the role of insulin resistance in the reversal of NAFLD at short and long terms.

To date, the association between weight loss and improvements in NAFLD as subject of research reveals no single intervention beyond weight loss to promote effective improvements in the outcomes of NAFLD patients (39, 40). In 2015, a prospective uncontrolled study of 293 patients undergoing calorie restriction and lifestyle changes for 52 weeks, with paired liver biopsies, found that a relative loss of 7–10% body weight improved NAFLD activity score in 88–100% of patients and resolved steatohepatitis in 84–90% of patients (41). Accordingly, clinical practice guidelines recommend a weight loss of at least 7% aiming to achieve histologic improvement in steatohepatitis and necroinflammation (42). Regrettably, long-term shift in dietary and lifestyle behavior is challenging, with only one in five patients undergoing conventional lifestyle intervention successfully achieving weight loss goals, reflecting the low adherence and reduced efficacy of most interventions (43). Alternatively, bariatric surgery has shown to be effective in reducing VAT and liver fat fraction, however, at the cost of reducing lean mass and at higher rates of complications, which make this approach unsuitable for wide clinical application (13–15). As shown in our and other studies, VLCKD may be a safe and effective short-term approach to achieve a rapid reduction in VAT and liver fat content, before patients are transitioned to the next steps of weight loss and NAFLD treatment, the latter focusing on lifestyle modifications and long-term maintenance (17, 44, 45). Additionally, the short-term positive results seen with VLCKD may act as an ally to increase adherence to treatment. In the clinical setting, an unquestionable positive response to treatment perceived in short periods of time often acts as a spur to increase adherence even in the more resistant patients (46, 47).

Although other studies have investigated VLCKD as an effective approach for reversing NAFLD in patients with obesity, the sparsity of studies with accurate measurements of liver outcomes has been highlighted by experts as a major limitation to confirm this hypothesis (44). Hence, to further contribute to the understanding of this process, we designed a study using MRI for the measurements of outcomes, which has been proven the most reliable and accurate non-invasive method for fat quantification (19, 21, 48). VLCKDs represent a nutritional intervention that mimics fasting through a marked restriction of daily carbohydrate intake (44). Accordingly, the commercially available weight loss program used in our study (Pronokal® Method) is a multidisciplinary method that uses a VLCKD relying on <50 g of carbohydrates per day and high-biological-value proteins, the latter intended to prevent the loss of lean mass (16), in addition to physical activity and individual supportive counseling. This method has been shown to result in effective weight loss, with low risks and high adherence (24). Although concerns of liver-related complications associated with rapid weight loss have been mentioned in the literature (46), neither alterations in markers of liver function nor changes in liver stiffness were observed in our study.

None of the groups in our study showed signs of advanced liver fibrosis based on MR elastrography results. Although our sample size was small, this should be related to the low prevalence of liver fibrosis in the general population. Also, similar to others (17), there were no significant changes in liver stiffness during treatment. Of note, at baseline, two patients in the LC group and one in the VLCKD group had liver stiffness values at the upper limit for absence of fibrosis considered in this study (2.8 kPa). Although stiffness values between 2.5 and 2.8 kPa have been proposed as potential indicator of liver inflammation (22, 27, 28), these studies were performed in different populations, and therefore, results cannot be directly translated to our cohort. Further studies should be performed to elucidate the clinical relevance of these values in patients with NAFLD and obesity.

Some limitations of our study should be listed. First, while both groups were formed by individuals undergoing weight-loss treatment for clinical care, our sample size is small and with a predominance of non-diabetic female patients, which should warrant caution when extrapolating our results to other populations. Further studies with larger sample sizes should be encouraged to validate our results. Second, although volumetric MRI methods could have been used to quantify VAT, the limited availability and high costs and complexity associated with these techniques restrict their application in clinical practice (49). Finally, we did not assess blood ketone levels, which could have provided insights into the mechanisms underlying the VLCKD-mediated effects on VAT and liver fat reduction.

In conclusion, patients undergoing a VLCKD program achieved superior weight loss, with significant VAT and liver fat fraction reductions when compared to a standard LC diet. These results corroborate other studies indicating that the rapid mobilization of liver fat demonstrated with VLCKD could serve as an effective short-term alternative for the treatment of NAFLD. A dietary plan with very low caloric and very low carbohydrate content as part of a multidisciplinary approach to lose weight could be the first step to positively reset the lipid metabolism in patients with NAFLD due to obesity, coupled with subsequent long-term maintenance period, according to the recommendations in VLCKD interventions.

## Data Availability Statement

The raw data supporting the conclusions of this article will be made available by the authors, without undue reservation, to any qualified researcher.

## Ethics Statement

The studies involving human participants were reviewed and approved by Comitê de Ética em Pesquisa do Instituto Estadual de Diabetes e Endocrinologia. The patients/participants provided their written informed consent to participate in this study. This trial has been registered at clinicaltrials.gov under Identifier NCT04322110.

## Author Contributions

The Pronokal personnel (IS and GG), were involved in the study design and revised the final version of the manuscript, without intervention in the analysis of data, statistical evaluation, and final interpretation of the results of this study. All other authors contributed to the article and approved the submitted version.

## Conflict of Interest

GG and IS are employees of Medical Department of Pronokal Spain. The remaining authors declare that the research was conducted in the absence of any commercial or financial relationships that could be construed as a potential conflict of interest.

## Footnotes

^1^http://www.fao.org/3/a-y5686e.pdf (accessed on November, 16, 2016)

^2^https://www.who.int/nutrition/publications/nutrientrequirements/WHO_TRS_935/en/ (accessed on November 16, 2016)
