# Supplementary material for: Efficacy of a 2-Month Very Low-Calorie Ketogenic Diet (VLCKD) Compared to a Standard Low-Calorie Diet in Reducing Visceral and Liver Fat Accumulation in Patients With Obesity
Source: Front Endocrinol (Lausanne). 2020 Sep 14;11:607. doi: 10.3389/fendo.2020.00607 (PMC7521128; doi:10.3389/fendo.2020.00607)
Supplement: Supplementary file 1 [file Table_1.docx]

**Supplementary Materials**

Throughout the duration of the study, patients undergoing the ketogenic diet received the following daily supplements of vitamins and minerals: Vitamin A, 600 mcg; Vitamin D, 5.0 mcg; Vitamin E, 10mg; Vitamin B1, 1.2 mg; Vitamin B2, 1.3mg; Vitamin B6, 1.3mg; Vitamin C, 45 mg; Vitamin B5, 5.0mg; Nicotinamide, 16mg; Folic acid, 240 mcg; Biotin 30 mcg; Vitamin B12, 2.4 mcg; Vitamin K, 65 mcg; Iron, 14mg; Iodine, 130 mcg; Manganese, 2.3mg; Selenium, 34 mcg and trace elements (Sodium: 1g/d, Potassium:1.6 g/d, Calcium: 800 mg/d; Magnesium: 375 mg/d).
